# Acute flaccid myelitis in a polio-like syndrome

**DOI:** 10.1590/0037-8682-0259-2020

**Published:** 2020-11-13

**Authors:** Ronaldo Gonçalves Pereira, Bernardo Carvalho Muniz, Bruno Niemeyer de Freitas Ribeiro

**Affiliations:** 1 Hospital Casa de Portugal, 3D Diagnóstico por Imagem, Rio de Janeiro, RJ, Brasil.; 2 Instituto Estadual do Cérebro Paulo Niemeyer, Departamento de Radiologia, Rio de Janeiro, RJ, Brasil.; 3 Grupo Fleury, São Paulo, SP, Brasil.

**FIGURE 1:**
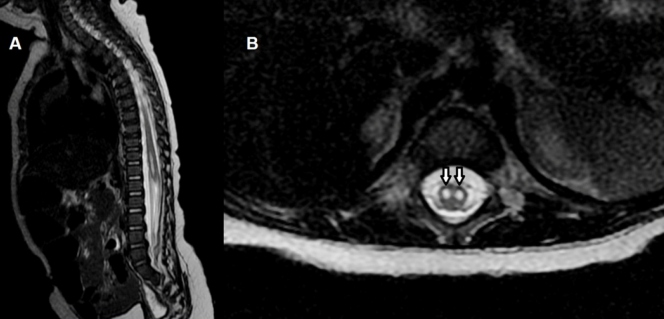
Magnetic resonance imaging of the spine, T2 sagittal **(A)** and T2 axial **(B)**. Bilaterally symmetric circular to ovoid foci of high T2-weighted signals in the anterior horn cells of the spinal cord compatible with the “snake eye” sign (arrows in B).

A previously healthy 5-year-old boy was admitted for fever, vomiting, and dyspnea, evolving with masticatory automatism, areflexia, and asymmetric flaccid tetraparesis, with preserved sensitivity. Magnetic resonance imaging (MRI) of the spine showed areas with symmetrical T2 hypersignals bilaterally in the anterior horns of the spinal cord, without contrast enhancement, in a “snake eye” sign (Figure 1).

Lumbar puncture identified anti-Epstein Barr virus (EBV) IgG and IgM antibodies in the cerebrospinal fluid.

Acute flaccid myelitis, an inflammation of the spinal cord that usually occurs after a viral disease, is characterized by flaccid paralysis of one or more members and may be associated with back pain, decreased sensitivity, and dysfunction of cranial nerves^1^ ^,^ ^2^. Poliovirus was the most common cause before widespread vaccination in the 1950s. Currently, other enteroviruses are primarily responsible, such as Coxsackie and enterovirus 71. MRI typically shows spinal cord involvement in the anterior horns of the gray substance (“snake eye” sign), affecting one or more spine segments.

EBV is the etiological agent of infectious mononucleosis, generally a benign and self-limiting disease, capable of causing complications in the central nervous system, such as encephalitis, cerebellitis, polyradiculomyelitis, cranial and peripheral neuropathies and myelitis, the latter being rare but the most common presentation in children^3^. Neuroimaging findings on MRI are characterized by hyperintense T2-weighted lesions, and involvement of the anterior medullary horns may be present, with a “snake eye” sign.

Treatment is with antivirals, such as acyclovir, and corticosteroids^3^. Most patients survive, but permanent neurological deficit and death can occur.
